# A Benign Case of Hepatic Gas

**DOI:** 10.5811/cpcem.2018.11.40815

**Published:** 2019-01-07

**Authors:** Lawrence K. Hou, Sheryl L.C. Diaz, Deborah L. Kimball

**Affiliations:** * Touro College of Osteopathic Medicine, Middletown, New York; † Stanford University School of Medicine, Department of Emergency Medicine, Palo Alto, California

## CASE PRESENTATION

A 33-year-old obese male with a history of well-controlled type II diabetes and hyperlipidemia presented to the emergency department with a one-day history of recurrent non-bloody diarrhea and abdominal pain in the morning progressing to significant nausea, increased non-radiating abdominal pain, and multiple episodes of non-bilious, non-bloody emesis in the evening. The patient reported 8/10 non-radiating, sharp, epigastric abdominal pain upon arrival. Physical examination findings revealed tenderness and rigidity in the right lower quadrant. The patient had an initial white blood cell count of 22.9 cells/millimeter^3^ (mm^3^) anion gap of 16 milliequivalents per liter (L), glucose level of 203 millimoles per liter (mmol/L), and a lactate of 3.01mmol/L. A computed tomography (CT) of abdomen and pelvis with intravenous contrast showed a mild wall thickening of the terminal ileum with multiple reactive mesenteric lymph nodes in the right lower quadrant indicative of inflammation, and a small volume of hepatic gas in the left hepatic lobe (Image). Point-of-care ultrasound of the abdomen confirmed the presence of hepatic gas in the left hepatic lobe (Video). The patient received two L of normal saline and was reevaluated showing significant pain relief. Ciprofloxacin, metronidazole, and vancomycin were given to treat an infectious etiology causing terminal ileum inflammation and diarrhea. The patient was admitted with gastroenteritis and subsequently discharged.

## DISCUSSION

Hepatic portal venous gas is commonly associated with mesenteric ischemia with a mortality rate of 75%.^1^,^2^ Mirmanesh et al. described a similar presentation and management in an older patient with type II diabetes mellitus diagnosed with viral gastroenteritis.^3^ Modern advancements in high-resolution CT and ultrasound are leading to increased recognition of benign cases of hepatic portal venous gas.^4^ This information should prompt emergency physicians to be more aware of benign etiologies that can cause hepatic gas.

**CPC-EM Capsule**

What do we already know about this clinical entity?

*The presence of hepatic gas is a serious clinical finding associated with diseases of a high mortality rate. Benign causes of hepatic gas remain rare in literature*.

What is the major impact of the image(s)?

*Hepatic gas associated with a benign diagnosis of gastroenteritis was found using point-of-care ultrasound and computed tomography in the emergency department setting*.

How might this improve emergency medicine practice?

*Emergency physicians may now be more aware of benign etiologies causing hepatic gas, using routinely practiced emergency medicine imaging modalities*.

## Supplementary Information

Video Ultrasound clip showing hyperechoic regions indicative of hepatic portal venous gas (arrows).

## Figures and Tables

**Image:**
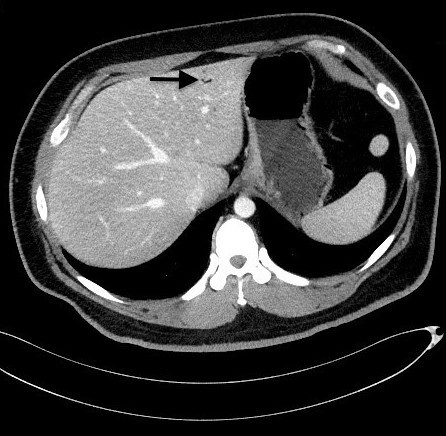
Computed tomography with contrast of abdomen shows hepatic portal venous gas in the anterior portion of the left hepatic lobe (arrow).

## References

[1] Liebman PR, Patten MT, Manny J (1978). Hepatic-portal venous gas in adults: etiology, pathophysiology and clinical significance. Ann Surg.

[2] Kinoshita H, Shinozaki M, Tanimura H (2001). Clinical features and management of hepatic portal venous gas: four case reports and cumulative review of the literature. Arch Surg.

[3] Mirmanesh M, Nguyen QS, Markelov A (2013). A case of hepatic portal venous gas due to viral gastroenteritis. Hepat Med.

[4] Shah PA, Cunningham SC, Morgan TA (2011). Hepatic gas: widening spectrum of causes detected at CT and US in the interventional era. Radiographics.

